# Chromosome loci vary by juvenile myoclonic epilepsy subsyndromes: linkage and haplotype analysis applied to epilepsy and EEG 3.5–6.0 Hz polyspike waves

**DOI:** 10.1002/mgg3.195

**Published:** 2016-01-23

**Authors:** Jenny E. Wight, Viet‐Huong Nguyen, Marco T. Medina, Christopher Patterson, Reyna M. Durón, Yolly Molina, Yu‐Chen Lin, Iris E. Martínez‐Juárez, Adriana Ochoa, Aurelio Jara‐Prado, Miyabi Tanaka, Dongsheng Bai, Sumaya Aftab, Julia N. Bailey, Antonio V. Delgado‐Escueta

**Affiliations:** ^1^ Epilepsy Genetics/Genomics LaboratoriesVA GLAHS – West Los AngelesLos AngelesCalifornia; ^2^ GENESS International ConsortiumLos AngelesCalifornia; ^3^ National Autonomous University of HondurasTegucigalpaHonduras; ^4^ Universidad Tecnológica Centroamericana (UNITEC)TegucigalpaHonduras; ^5^ Department of NeurologyDavid Geffen School of Medicine at UCLALos AngelesCalifornia; ^6^ National Institute of Neurology and NeurosurgeryMexico CityMexico; ^7^ Department of EpidemiologyFielding School of Public Health at UCLALos AngelesCalifornia

**Keywords:** Absence, electroencephalogram, endophenotype, epilepsy, juvenile myoclonic, linkage, pyknolepsy

## Abstract

Juvenile myoclonic epilepsy (JME), the most common genetic epilepsy, remains enigmatic because it is considered one disease instead of several diseases. We ascertained three large multigenerational/multiplex JME pedigrees from Honduras with differing JME subsyndromes, including Childhood Absence Epilepsy evolving to JME (CAE/JME; pedigree 1), JME with adolescent onset pyknoleptic absence (JME/pA; pedigree 2), and classic JME (cJME; pedigree 3). All phenotypes were validated, including symptomatic persons with various epilepsies, asymptomatic persons with EEG 3.5–6.0 Hz polyspike waves, and asymptomatic persons with normal EEGs. Two‐point parametric linkage analyses were performed with 5185 single‐nucleotide polymorphisms on individual pedigrees and pooled pedigrees using four diagnostic models based on epilepsy/EEG diagnoses. Haplotype analyses of the entire genome were also performed for each individual. In pedigree 1, haplotyping identified a 34 cM region in 2q21.2–q31.1 cosegregating with all affected members, an area close to 2q14.3 identified by linkage (*Z*_max_ = 1.77; pedigree 1). In pedigree 2, linkage and haplotyping identified a 44 cM cosegregating region in 13q13.3–q31.2 (*Z*_max_ = 3.50 at 13q31.1; pooled pedigrees). In pedigree 3, haplotyping identified a 6 cM cosegregating region in 17q12. Possible cosegregation was also identified in 13q14.2 and 1q32 in pedigree 3, although this could not be definitively confirmed due to the presence of uninformative markers in key individuals. Differing chromosome regions identified in specific JME subsyndromes may contain separate JME disease‐causing genes, favoring the concept of JME as several distinct diseases. Whole‐exome sequencing will likely identify a CAE/JME gene in 2q21.2–2q31.1, a JME/pA gene in 13q13.3–q31.2, and a cJME gene in 17q12.

## Introduction

According to the World Health Organization (2014), “Epilepsy is the most common serious brain disorder worldwide with no age, racial, social class, national nor geographic boundaries”. The epilepsies afflict approximately 65 million persons globally, and almost 3 million in the USA (Hauser and Hesdorffer 1990; England et al. 2012). Idiopathic generalized epilepsies, now called genetic generalized epilepsies (Berg et al. 2010) comprise at least 40% of these epilepsies in the USA and 8% in Central America, including Honduras (Obeid and Panayiotopoulos 1988; Jallon 1997; Nicoletti et al. 1999; Medina et al. 2005). Juvenile myoclonic epilepsy (JME) is the most common of these genetic generalized epilepsies and accounts for 18% of the genetic epilepsies and 11.9% of all epilepsies (Goosses 1984; Camfield et al. 2013), thus afflicting approximately 8.5 million persons worldwide and about 360,000 persons in the USA.

The “sine qua non” in the diagnosis of JME are the myoclonic seizures or “myoclonias”. French authors established the link between these myoclonias and epilepsy in the mid nineteenth century. In 1854, Delasiauve proposed the term “motor petit mal” to describe myoclonic jerks. Herpin (1867), gave the first reliable description of JME when he wrote about “impulsions” or “commotions” or “secousses” or “shocks that shake the whole body like an electric commotion” … shocks which were violent enough to cause a patient to involuntarily throw an object from their hands. Rabot (1899) introduced the term “myoclonic” in his description of a patient with “myoclonic shocks”, in which the clinical description was equivalent to Herpin's shocks and Delasiauve's “motor petit mal”.

Aside from the myoclonic seizure phenotype, a more precise definition for the JME syndrome and the awakening morning myoclonias was not provided until two separate groups, one writing in German and the other in Spanish reported cohorts of JME patients. Janz and Christian writing in German in 1957, described JME in 47 patients as “impulsiv petit mal.” Castells and Mendilaharsu; writing in Spanish used the term “bilateral and conscious myoclonic epilepsy” to describe JME in 70 patients, first in a doctoral thesis in 1954 and 1956and then in 1958 in a Latin American journal. Lund et al. (1976), was the first to name the disorder JME. It was 1984 when the first ictal video‐EEG recordings of myoclonic seizures and clonic‐tonic‐clonic grand mal convulsions showed 3.5–6.0 Hz polyspike waves (Delgado‐Escueta and Enrile‐Bacsal 1984). In 1989, the International League Against Epilepsy (ILAE) asserted that Janz's “impulsiv petit mal” was equivalent to Lund's “JME,” and classified the disorder as a form of idiopathic generalized epilepsy. In 2010, JME was classified by the ILAE as a genetic generalized epilepsy (Berg et al. 2010).

During the six decades following the landmark papers by Castells and Mendilaharsu, and by Janz and Christian, JME emerged as an enigmatic epilepsy (Koepp et al. 2014); multifocal frontocortical‐subcortical networks revealed by advanced MRI imaging questioned whether JME was truly a primary generalized form of epilepsy (Woermann et al. 1999; Kim et al. 2007; Alhusaini et al. 2013), and varying course of illness with lifelong persistence versus early remission of seizures reported in cohorts from different continents (Camfield and Camfield 2009; Senf et al. 2013) suggested that JME might be composed of several diseases. Hence, as early as 1988, there were efforts to identify genes that underlie the JME disease using protein markers during linkage analyses. Greenberg and UCLA colleagues (1988) first reported genetic linkage of JME and its 3.5–6.0 Hz polyspike wave EEG pattern in 11 small families to the properdin BF factor and the human leukocyte antigen complex on chromosome 6p21.2. Subsequent studies in JME families from Los Angeles, Belize, Mexico, Honduras, and Germany used microsatellites during linkage analysis and showed separate loci in chromosome regions 6p21.2, 6p20, and 6p12 (Durner et al. 1991; Weissbecker et al. 1991; Suzuki et al. 2004). Ultimately, further genetic, informatics and molecular experimental studies identified mutations in Myoclonin1*/EFHC1* in 6p12 as disease causing in JME (Suzuki et al. 2004; De Nijs et al. 2012). To date, 29 chromosomal loci in chromosomes 2, 3, 5, 6, 7, 10, 13, 15, 16, 18, and 19 have been genetically linked with JME (Koepp et al. 2014). Mutations have been identified in five genes (*CACNB4*, [HGNC:1404], *CASR* [HGNC:1514], *GABRA1* [HGNC:4075], *GABRD* [HGNC:4084], and Myoclonin1*/EFHC1* [HGNC:16406]) (Delgado‐Escueta 2007; Delgado‐Escueta et al. 2013). Additionally, three single‐nucleotide polymorphisms (SNPs) believed to be susceptibility alleles in bromain domain‐containing protein‐2 (*BRD2* [HGNC:1103]), Connexin 36 (*Cx‐36*) also called GJD2 (Gap Junction protein Delta2) (HGNC:19154), and malic enzyme 2 (*ME2*) (HGNC:6984) and microdeletions in 15q13.3, 15q11.2, and 16p13.11 have also been identified and may contribute risk to JME (Delgado‐Escueta 2007; Helbig et al. 2010; Delgado‐Escueta et al. 2013). These five Mendelian JME genes listed above account, in the least, for rare and scarce families with private mutations, with the exception of EFHC1, which accounts for at most 3–20% of JME cohorts (Stogmann et al. 2006; Annesi et al. 2007; Medina et al. 2008; Jara‐Prado et al. 2012), leaving the majority fraction (80–97%) of JME without an identified genetic defect. Thus, the quest for disease‐causing variants in JME continues.

Here, we performed linkage and haplotype analysis on three large (>30 members) multigeneration families from Honduras ascertained through a JME proband, each exhibiting a different subsyndrome of the disease, namely, Childhood Absence Epilepsy evolving to JME (CAE/JME) in pedigree 1, JME with adolescent onset pyknoleptic absence (JME/pA) in pedigree 2, and classic JME (cJME) in pedigree 3. In each pedigree, at least seven other family members were diagnosed with a form of generalized epilepsy or were clinically asymptomatic but demonstrated EEG diffuse 3.5–6.0 Hz polyspike waves.

## Subjects and Methods

### Ethical compliance

The study was approved by the Medical Institutional Review Boards for the David Geffen School of Medicine at UCLA, National Institute of Neurology and Neurosurgery in Mexico City, Mexico, and the National Autonomous University of Honduras. Informed consent was obtained for all participants, patients or from parents of epilepsy‐affected or unaffected children.

### Patient and family ascertainment

#### Probands

The probands and their families were collected by neurologists at the local study sites of the international consortium of Genetic Epilepsy Studies (GENESS). Diagnosis of JME subsyndromes is based on the work of Martínez‐Juárez et al. (2006). Diagnosis of JME in general is based on the 1989 and 2010 Commission and Classification of the Epilepsies by the ILAE and the 2010 Avignon workshop on JME (ILAE, 1989; Berg et al. 2010; Kasteleijn‐Nolst Trenité et al. 2013).

The proband of pedigree 1 with CAE/JME presented at 10 years of age with pyknoleptic (at least daily) absences with 3‐4 Hz spike and wave complexes. She developed myoclonic seizures and clonic‐tonic‐clonic and tonic‐clonic seizures with EEG 4–6 Hz polyspike wave complexes during menarche at 12 years of age. At 32 years of age, absences occurred at 5 per day while convulsions appeared at 3 to 4 am, 4 days before menses, during ovulation, or when fatigued or sleep deprived.

The proband of pedigree 2 with JME/pA presented at 13 years of age with myoclonic, and tonic‐clonic and clonic‐tonic‐clonic seizures. Pyknoleptic absences started at 18 years of age with 3–5 Hz single spike and slow wave complexes.

The proband of pedigree 3 with cJME experienced frequent, severe, awakening myoclonic seizures of shoulders and arms, forearms, hands, and infrequently legs, without loss of consciousness at 14 years of age. Grand mal clonic‐tonic‐clonic or tonic‐clonic seizures were also present (Durón et al. 2005; Martínez‐Juárez et al. 2006) and spanioleptic (infrequent) absences rarely appeared.

The probands of pedigrees 1, 2, and 3 had normal neurological examinations and intelligence and normal computerized tomography of brain (Serratosa et al. 1996; Berg et al. 2010; Kasteleijn‐Nolst Trenité et al. 2013).

#### Family members and their EEGs

Families were extended to form multigenerational pedigrees. In order to determine who is affected among the family members, all living relatives willing to participate in each of the three pedigrees had 30‐min video‐electroencephalographs (VEEGs) and were interviewed and examined by a GENESS neurologist. Probands of pedigrees 1 and 2 had 24‐h ambulatory EEG and video recordings. When asymptomatic relatives had abnormal diffuse 3.5–6.0 Hz polyspike waves, further clinical examinations were performed and video‐EEG recordings confirmed the absence of clinical seizures. All members were studied by a (validation) team composed of at least two GENESS neurologists to confirm diagnosis, and VEEG results were read independently and blindly by two investigators. Blood samples were collected from each living and participating member.

Members were considered to have the EEG polyspike wave trait when they were clinically asymptomatic but had evidence of the fast variety (3.5–6.0 Hz) polyspike wave or spike wave complexes. The EEG of affected members have also displayed diffuse, bilateral, synchronous 2.0–3.0 Hz single spike and slow wave complexes or more irregular diffuse 4.0–6.0 Hz sharp and slow waves mixed with random spikes along with the fast variety of spike wave complexes (Serratosa et al. 1996). Members were considered unknown if they had an unconfirmed diagnosis of epilepsy. Members were considered unaffected if they did not meet any of the above mentioned criteria.

Nonproband family members of these three pedigrees were diagnosed with JME, or generalized clonic‐tonic‐clonic or tonic‐clonic grand mal seizures only (GM) as the sole clinical phenotype, or generalized clonic‐tonic‐clonic or tonic‐clonic grand mal seizures plus absence seizures, or absence seizures/epilepsy only, absence seizures/epilepsy with eyelid myoclonia, or a combination of these generalized epilepsies (Durón et al. 2005).

### Pedigree structure and their family members

**Figure 1:**
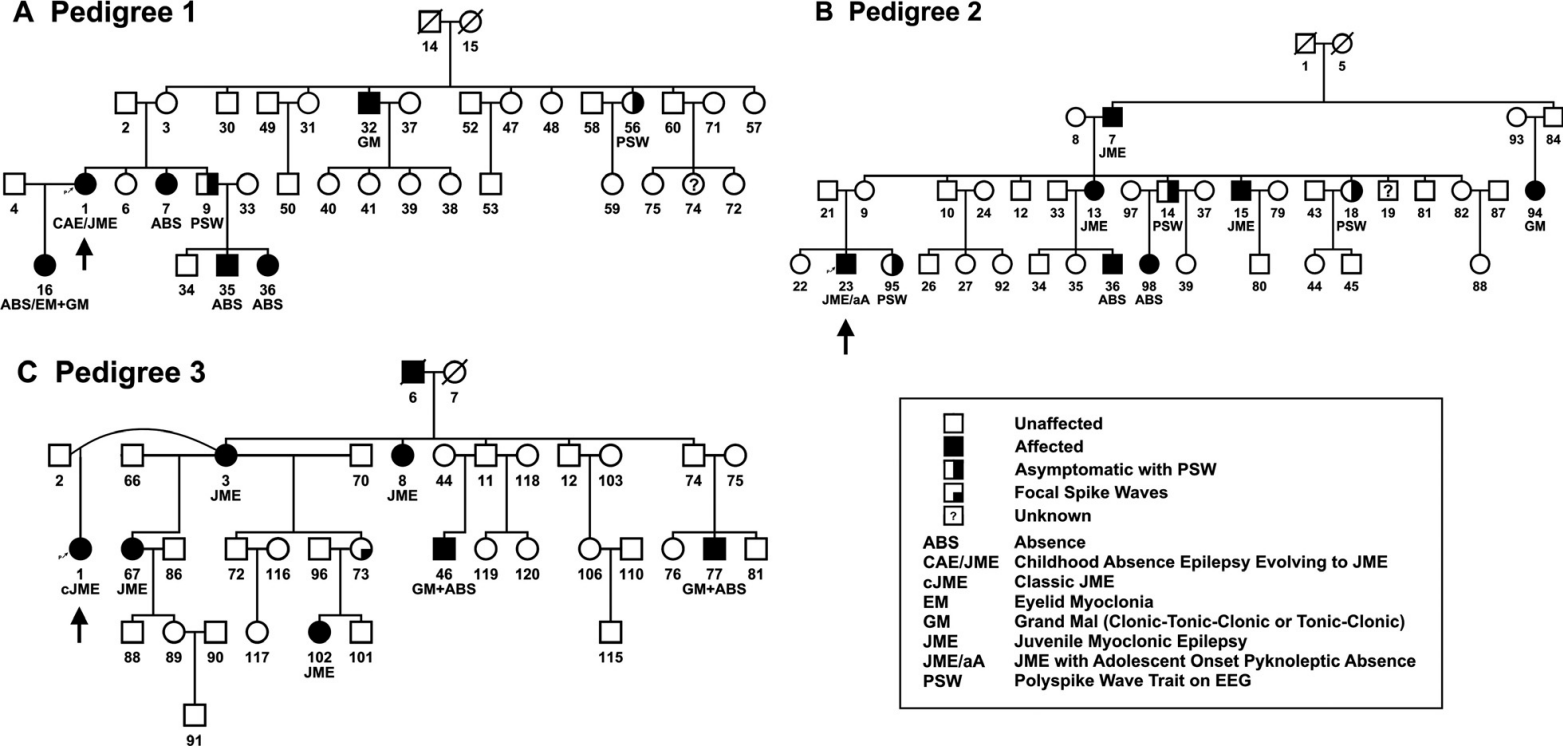
Pedigree structure and phenotypes of the families studied. (A) Pedigree 1 was ascertained through a proband with CAE evolving to JME. (B) Pedigree 2 was ascertained through a proband with JME with adolescent onset pyknoleptic absence. (C) Pedigree 3 was ascertained through a proband with classic JME. All three families are from Honduras.

**Figure 2:**
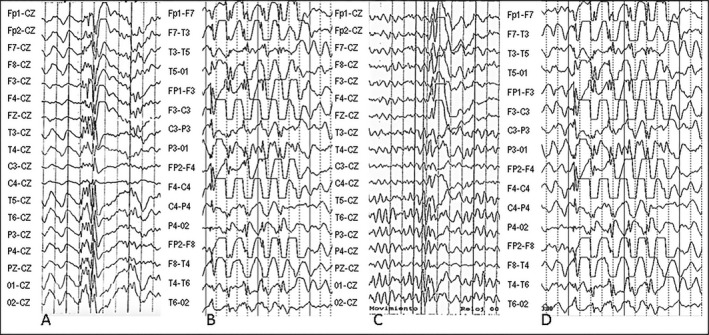
EEGs from pedigrees evaluated. Pedigree 1: (A) Female proband (member 1) 31 years old with CAE evolving to JME had ambulatory EEG which detected bursts of isolated or repetitive 4–6 Hz polyspike and slow waves (PSW) on awakening; (B) Daughter of proband (member 16) was 6 years old with absences, tonic‐clonic seizures (TC) and eyelid myoclonias and had bursts of isolated or repetitive 3 Hz single spike and slow waves (SW); Pedigree 2: (C) Male proband (member 23) 22 years old had isolated or repetitive 5–6 Hz PSW and myoclonias during the resting state; (D) Female maternal cousin (member 98) 10 years old had clinical absences and diffuse 3–4 Hz PSW and SW complexes spontaneously or during hyperventilation. Speed was 10 sec/page, sensitivity was 10 uV/mm for the EEGs.

Honduras pedigree 1 (Fig. 1A) is comprised of four generations and 37 members, 28 of whom were genotyped. The proband is affected with CAE evolving to JME (member 1 in Fig. 2A), three members are diagnosed with absence epilepsy only (members 7, 35, 36), one with childhood absence plus eyelid myoclonia and generalized clonic‐tonic‐clonic or tonic‐clonic grand mal seizures (member 16 in Fig. 2B), and one with generalized clonic‐tonic‐clonic or tonic‐clonic grand mal seizures only (member 32). Additionally, two members are clinically asymptomatic with the EEG polyspike wave trait (members 9, 56) and one member is considered unknown because she was reported to have had two tonic‐clonic seizures but had normal EEGs and remained free of seizures even without treatment (member 74). The remaining members are unaffected.

Honduras pedigree 2 (Fig. 1B) is comprised of four generations and 40 members, with 32 of the members genotyped. The proband is affected by JME with adolescent onset pyknoleptic absence (member 23 in Fig. 2C). Three other members are affected with JME (members 7, 13, 15), two members with absence epilepsy only (members 36, 98; Fig. 2D), one member with generalized clonic‐tonic‐clonic or tonic‐clonic grand mal seizures only (member 94), and three members are clinically asymptomatic but exhibit the EEG polyspike wave trait (members 14, 18, 95). Two individuals in this pedigree are considered unknown because they exhibited phenotypes that could not be confirmed as epilepsy, having fainting spells and abnormal nonspecific EEGs with nocturnal terrors (members 19, 44, respectively). The remaining members are unaffected.

Honduras pedigree 3 (Fig. 1C) is comprised of 39 members in five generations, 21 of whom were genotyped. The proband is affected with classic JME (member 1). Four other members are also affected with JME (members 3, 67, 99, 102) and three with generalized clonic‐tonic‐clonic or tonic‐clonic grand mal seizures plus adolescent onset absence (members 8, 46, 77). One deceased member (member 6), whose blood was unavailable for genotyping and therefore was not included in linkage or haplotype analyses, had a diagnosis of generalized clonic‐tonic‐clonic or tonic‐clonic grand mal seizures based on a reliable history from family members, though this was unconfirmed by GENESS neurologists. The remaining family members in this pedigree were classified as unaffected. There were no clinically asymptomatic persons who carried the EEG polyspike wave trait in this pedigree.

### SNP genotyping

DNA was extracted from whole blood using the QIAamp DNA Blood Mini Kit or the QIAamp DNA Blood Midi Kit (Qiagen, Valencia, CA) following the manufacturer's protocols. At least 25 μL of DNA were obtained at a concentration of 50–100 ng/μL. Genotyping services were provided by the Center for Inherited Disease Research (CIDR). Using a custom resynthesis of the InfiniumLinkage‐24 marker panel, genotyping of 5208 SNPs were attempted and 5185 SNPs (99.6%) were released. SNPs were called using GenomeStudio version 2011.1, Genotyping Module 1.9.4, Gentrain version 1.0. The Mendelian consistency rate of these pedigrees was 99.987% and the concordance rate was 99.81%.

### Linkage analysis

We developed four diagnostic models for purposes of linkage analysis: Model #1: all family members with any form of clinically symptomatic epilepsy were considered affected; Model #2: all family members with any form of clinically symptomatic epilepsy plus clinically asymptomatic members with polyspike waves evidenced on their EEGs were considered affected; Model #3: only JME affected members were considered affected; and Model #4: all family members with JME plus clinically asymptomatic members with polyspike waves evidenced on their EEGs were considered affected. Linkage analyses were run on each of the individual pedigrees under each diagnostic model. Additionally, linkage analyses were also run on the combination of the three Honduran pedigrees under each diagnostic model.

Two‐point parametric linkage analysis was completed using Superlink v1.6 (Fishelson and Geiger 2002, 2004) and ELOD scores were simulated using FastSLink v2.51 in the EasyLinkage Plus v5.08 package (Ott 1989; Weeks et al. 1990). Pedigrees were analyzed using an autosomal dominant model with 70% penetrance, disease allele frequency of 0.01, and penetrance of the homozygote nondisease genotype of 0.001 in both the simulated and linkage analyses. LOD scores were calculated at 0.05 recombination increments from recombination values of 0.00 to 0.50, and simulated ELODs were calculated using 1000 replications and the same parameters previously mentioned for the two‐point parametric linkage analysis. A custom SNP map was provided by CIDR based on the SNPs included in the custom InfiniumLinkage‐24 marker panel. LOD scores of 2.4 or greater were considered to be suggestive for linkage while LOD scores of 3.3 or greater were considered to be significant for linkage (Lander and Schork 1994).

The pedigrees analyzed, similar to many previously described JME families, exhibit an incompletely penetrant autosomal dominant mode of inheritance with the possibility of sporadic cases due to new mutations (Delgado‐Escueta et al. 1994). Even if the model specifications were slightly incorrect, Clerget‐Darpoux et al. (1986) argued that “the power of the linkage test is sensitive to the degree of dominance, and slightly to the penetrance,” specifically that “misspecifying the penetrance leads to a slight underestimation of (LOD score)”. Additionally, the use of extended pedigrees and allowing for the possibility of sporadic cases, contains “a number of checks and balances, or “buffers,” from the viewpoint of linkage analysis” (Hodge and Greenberg 1992).

### Haplotype analysis

Using 5185 SNPs genotyped by CIDR, haplotypes were constructed for all chromosomes in each of the pedigrees by hand. For those regions which appeared to cosegregate and those regions in which there were multiple possible haplotypes, haplotypes were simulated using SimWalk v2.91, which imputes the haplotype with the highest probability of occurring. Chromosomal regions which cosegregated identically by descent in all affecteds of individual pedigrees were identified. Haplotypes exhibiting cosegregation were visualized using HaploPainter (v1.043, Slashdot Media, San Jose, CA, USA).

## Results

### ELOD scores

The highest average and maximum ELOD scores occurred when all pedigrees were combined under diagnostic model #2 (all family members with clinically symptomatic epilepsy plus clinically asymptomatic members with the EEG trait; average ELOD = 2.63 and maximum ELOD = 8.07; Table S1). In fact, regardless of which diagnostic model was run, the combination of the pedigrees produced a higher expected LOD score than any of the pedigrees could have produced on their own.

When individual pedigrees were run, the addition of the clinically asymptomatic members with the EEG trait to the affected pool in pedigrees 1 and 2 consistently produced a higher ELOD score than that produced when only clinically affected members were considered affected (model #2 scores greater than model #1; model #4 scores greater than model #3). This increase in ELOD score was particularly evident when comparing models #3 and #4 in pedigree 1, as only one family member in this pedigree was diagnosed with JME. The simulations using diagnostic models #3 and #4 produced smaller average and smaller maximum ELOD scores than those run under diagnostic model #1 and #2, particularly in pedigrees 2 and 3 as it counted less members as affected. In each pedigree and the combination of pedigrees, the results from diagnostic model #3 consistently produced the smallest scores, as it counted the fewest members as affected. Pedigree 3 has no clinically asymptomatic members with the EEG polyspike wave trait, therefore, the ELODs for this pedigree were unchanged between diagnostic models #1 and #2 and diagnostic models #3 and #4.

### Two‐point parametric linkage analysis

Linkage analyses were run under all four diagnostic models for Honduras pedigrees 1, 2, and 3 (genome wide plots in Fig. S1), as well as for the combination of the pedigrees (Table 1).

**Table 1:** Two‐point parametric linkage results for Honduran pedigrees

Diagnostic Model	Pedigree 1			Pedigree 2			Pedigree 3			All Pedigrees		
	Chr	SNP	*Z*_max_	Chr	SNP	*Z*_max_	Chr	SNP	*Z*_max_	Chr	SNP	*Z*_max_
#1. Clinically Affected	5q23.3	rs6871^a^	2.1101	4q35.2	rs996026	1.7451	9p22.3	rs1888952	2.5534	4q35.2	rs6553022	2.9525
	5q31.1	rs256332	2.0112	3p25.1	rs826423	1.7239	13q21.31	rs1553161	2.0414	5q21.3	rs1045706	2.8261
	5q31.3	rs29900	1.8016	4q35.1	rs830835	1.5961	18p11.22	rs1013785	1.8233	13q31.1	rs3127540	2.7527
	5q34	rs244903	1.7966	4q35.2	rs6553022	1.5747	13q14.13	rs4941527	1.8088	16p13.3	rs2301763	2.2292
	5q31.1	rs726847^a^	1.5808	16p13.3	rs2745165	1.5392	15q21.1	rs537848^a^	1.7938	5q33.1	rs357608	2.2155
#2. Clinically Affected + EEG PSW	2p23.2	rs2272386	2.5876	4q34.2	rs13132745	2.2576	9p22.3	rs1888952	2.5534	13q31.1	rs3127540	3.5040
	5q23.1	rs1469049	1.8863	4q35.1	rs907362	2.1289	13q21.31	rs1553161	2.0414	4q35.2	rs1869941	3.4578
	6q27	rs1886672	1.8250	4q34.2	rs1014381	2.0356	18p11.22	rs1013785	1.8233	16p13.3	rs1203974	3.3691
	4p15.1	rs890459	1.7304	13q21.31	rs1335686	1.9016	13q14.13	rs4941527	1.8088	16p13.3	rs8045185	2.9635
	2p12	rs3820749	1.7154	4q35.2	rs1869941	1.8815	15q21.1	rs537848^a^	1.7938	16p13.3	rs1211375	2.8545
#3. JME Affected	5q23.1	rs1469049	0.3757	6q22.1	rs1321813	1.8479	5q33.3	rs949602	1.3201	11q24.2	rs4627097	2.2380
	18q21.33	rs1943329	0.3752	11q24.2	rs2156449	1.7026	4q32.3	rs1566499	1.2756	5q21.1	rs2030605	1.6092
	17p13.1	rs1565816	0.3750	16q23.1	rs8062565	1.6970	4q28.1	rs2048266	1.1692	7q36.2	rs1657290	1.4950
	5q22.3	rs1496390	0.3747	11q24.2	rs4627097	1.6911	12p13.32	rs9300302	1.1671	6q15	rs2144363	1.4506
	5q22.2	rs1968557	0.3746	6q22.33	rs1543432	1.5131	5q34	rs32422	1.1656	6q16.2	rs2894891	1.4408
#4. JME Affected + EEG PSW	2q14.3	rs1028184	1.7656	6p22.3	rs760848	1.8265	5q33.3	rs949602	1.3201	4q28.3	rs1365372	2.1682
	8p22	rs1125265	1.7650	3p25.1	rs905946	1.7873	4q32.3	rs1566499	1.2756	3p23.1	rs826423	1.9816
	8p22	rs7841810	1.7645	4q35.1	rs907362	1.6256	4q28.1	rs2048266	1.1692	4q28.3	rs1499328	1.8648
	6q25.3	rs675162	1.7285	6p22.3	rs1355460	1.5999	12p13.32	rs9300302	1.1671	8p22	rs6991832	1.7302
	6q26	rs783182	1.6694	4q31.3	rs1516822	1.5636	5q34	rs32422	1.1656	8q22.3	rs614961	1.7219

LOD scores (*Z*_max_) were calculated for each diagnostic model under each pedigree and under the combination of pedigrees. Family members that were considered affected under each model are as follows: (model #1) all members affected with clinically symptomatic epilepsy, (model #2) all members affected with clinically symptomatic epilepsy and members with the EEG polyspike wave (PSW) trait, (model #3) all members affected with JME, and (model #4) all members affected with JME and members with the EEG PSW trait. Because pedigree 3 has no members with the EEG PSW trait, models #1 and #2 are identical, as are models #3 and #4. LOD scores were simulated using FastSLink v2.51 in the EasyLinkage Plus v5.08 package using an autosomal dominant model with 70% penetrance, disease allele frequency of 0.01, penetrance of the homozygote nondisease genotype of 0.001, and 0.05 recombination increments from recombination values of 0.00 to 0.50.

*θ*_m = f_ = 0.00 unless otherwise indicated.

a Indicates *θ*_m = f_ = 0.05.

#### Pedigree 1

The highest LOD obtained from all analyses was suggestive for linkage with *Z*_max_ (*θ*_m = f_ = 0.00) of 2.59 at rs2272386 on 2p23.2 under diagnostic model #2, which included all the epilepsy affected individuals (1 with CAE evolving to JME, 3 with absence only, 1 with absence and grand mal, 1 with grand mal only) and those with the EEG polyspike wave trait. A drop in *Z*_max_ occurred when asymptomatics with EEG polyspike waves were not considered affected (diagnostic model #1). Diagnostic model #3 produced a negligible LOD score in this pedigree because only one member has a JME diagnosis. Diagnostic model #4 produced LOD scores higher than those produced by diagnostic model #3 with *Z*_max_ (*θ*_m = f_ = 0.00) of 1.77 at rs1028184 on 2q14.3. This is a result of the addition of the clinically asymptomatic members with the EEG polyspike wave trait to the only person with JME.

#### Pedigree 2

The LOD score peaked at rs13132745 (*Z*_max_ = 2.26) on 4q34.2 when all forms of epilepsy (four individuals with JME, 2 with absence only, one with grand mal only) and three asymptomatic persons with the EEG polyspike wave trait were included as affected under diagnostic model #2. Aside from 4q34.2, the other locus identified by this model was 13q21.31 with *Z*_max_ (*θ*_m = f_ = 0.00) of 1.90 at rs1335686. Diagnostic model #3 produced LOD scores very similar to those produced under diagnostic models #1 and #4, though none of the same markers were seen among the top results of the three models and all failed to reach scores comparable to diagnostic model #2.

#### Pedigree 3

LOD scores were suggestive for linkage at rs1888952 on chromosome 9p22.3 under diagnostic models #1 and #2 (*Z*_max_ = 2.55), including all epilepsy affected members (five individuals with JME, three with grand mal and absence, one with grand mal only). Interestingly 13q21.31 was also identified by models #1 and #2 with *Z*_max_ (*θ*_m = f_ = 0.00) of 2.04 at rs1553161. Because no pedigree members were clinically asymptomatic with abnormal EEGs, the results for diagnostic models #1 and #2 are identical, as are the results for models #3 and #4.

#### Combined pedigrees

When the three Honduran pedigrees with JME proband diagnoses were combined, diagnostic model #2 produced LOD scores significant for linkage, and diagnostic model #1 produced LOD scores suggestive for linkage. When all clinically affected members and all clinically asymptomatics with EEG polyspike waves were included as affected under model #2, LOD score peaked at 3.50 at rs3127540 on 13q31.1. Under this analysis, rs1869941 at 4q35.2 and rs1203974 at 16p13.3 also reached the level of significance. When only the epilepsy affected members were considered affected under model #1, *Z*_max_ reached 2.95 at a second marker in 4q35.2 (rs6553022) and also had markers at 5q21.3 (rs1045706) and 13q31.1 (rs3127540) surpass the level suggestive for linkage. Similar to the individual pedigree analyses, the addition of the clinically asymptomatic members with EEG polyspike waves in the combined pedigree analysis increased LOD scores, crossing the threshold for significance.

### Haplotype analysis

**Figure 3:**
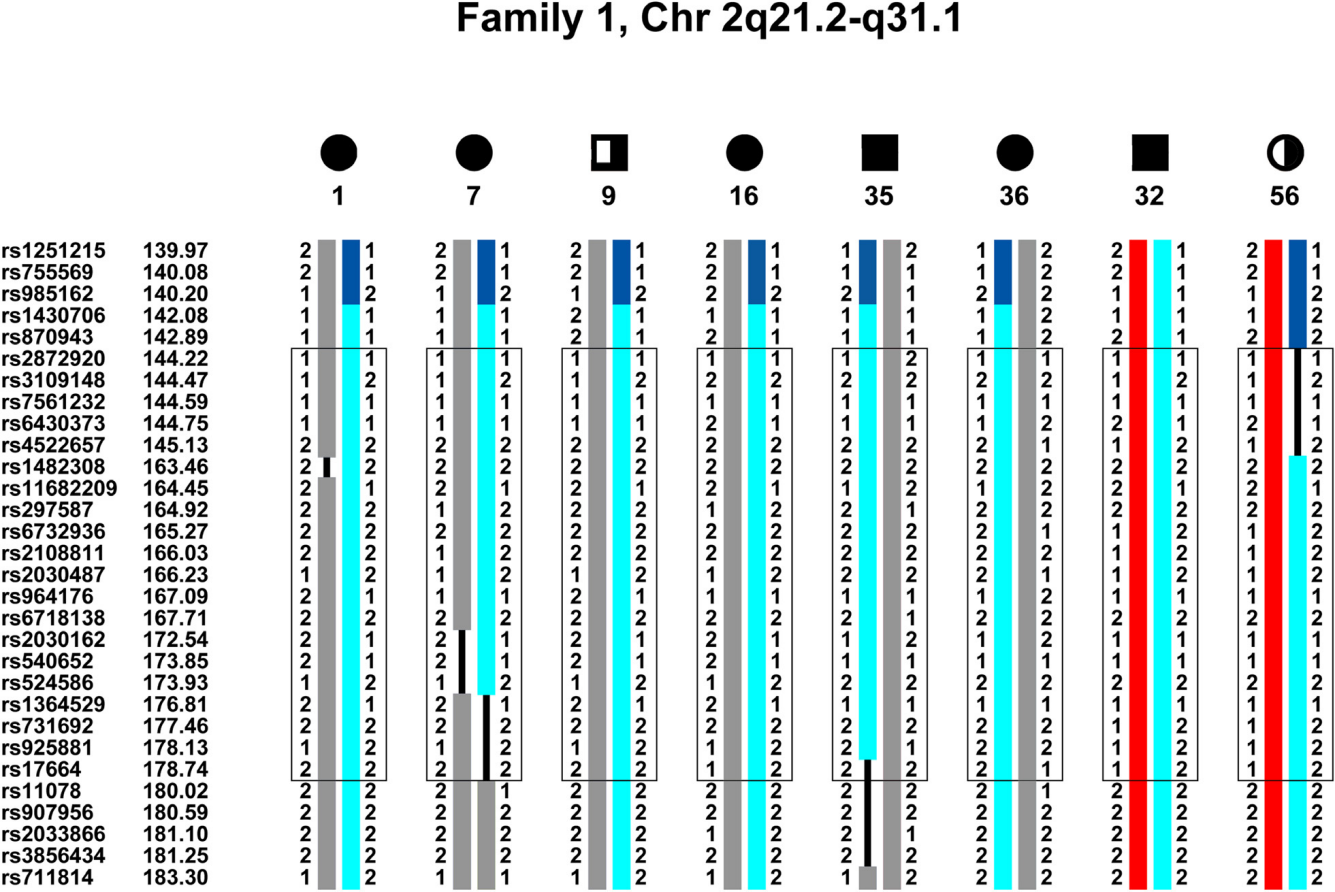
Haplotype of pedigree 1, chromosome 2q21.2–q31.1. An approximate 34 cM region from rs2872920 (144.22 cM) to rs17664 (178.74 cM) in chromosome 2q21.2–q31.1 cosegregates identically by descent (IBD) with all of the clinically and EEG affected members in the pedigree. It fails to cosegregate with member 74, the only individual whose phenotype is considered “unknown.” The cosegregating haplotype is in light blue. The upper boundary is demarcated by a recombination in member 56 and the lower boundary by a recombination in member 7. While all markers within the area were analyzed by SimWalk and cosegregate, markers between rs6430398 and rs1401750, and between rs889920 and rs1517342, as well as several markers in the represented region have been removed to condense the figure.

Haplotype analysis of the entire genome of pedigree 1 showed only one cosegregating chromosome region, namely a 34 cM area in 2q21.2–31.1, bordered by rs2872920 and rs17664, that cosegregates with all clinically affected persons and clinically asymptomatic individuals who carry the EEG polyspike wave trait (Fig. 3). This area is close to the 2q14.3 region exhibiting *Z*_max_ of 1.77. Chromosome loci 2p23.2 and 5q23, which had *Z*_max_ of 2.59 and 2.11, respectively, along with all other loci, are eliminated by haplotype analysis.

**Figure 4:**
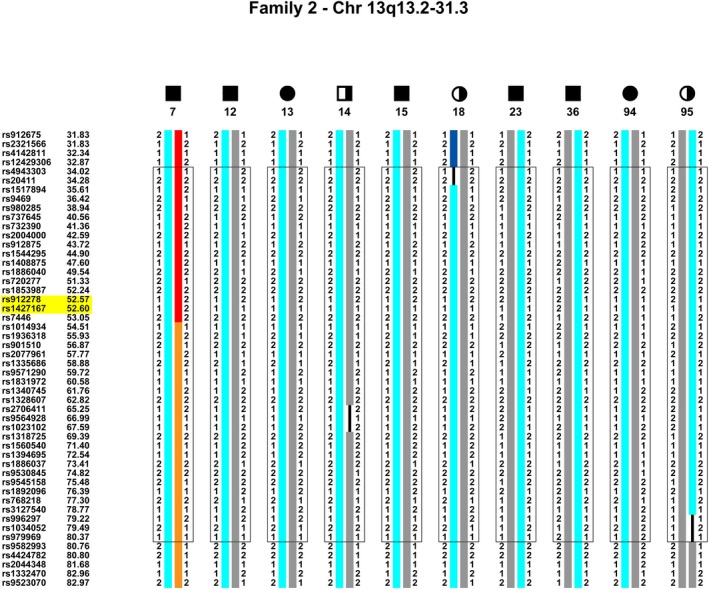
Haplotype of pedigree 2, chromosome 13q13.3–q31.2. An approximate 44 cM region from rs4943303 (34.02 cM) to rs979969 (80.37 cM) in chromosome 13q13.3–q31.2 cosegregates IBD with all of the clinically and EEG affected members in the pedigree. It fails to cosegregate with “unknown” members 19 and 44. The cosegregating haplotype is light blue. The upper boundary is demarcated by a recombination in member 18 and the lower boundary by a recombination in member 95. The small region of possible cosegregation in pedigree 3 within this region is demarcated with a yellow highlighted box. While all markers within the area were analyzed by SimWalk and cosegregate, markers within the region have been removed to condense the figure.

After haplotype analysis of the entire genome of pedigree 2, a 44 cM region bordered by rs4943303 and rs979969 in chromosome 13q13.3 to 13q31.2 cosegregates with all clinical and EEG affected members (Fig. 4). This is the only chromosome region to cosegregate in this pedigree and supports the 13q21.31 linkage area with *Z*_max_ of 1.9016.

**Figure 5:**
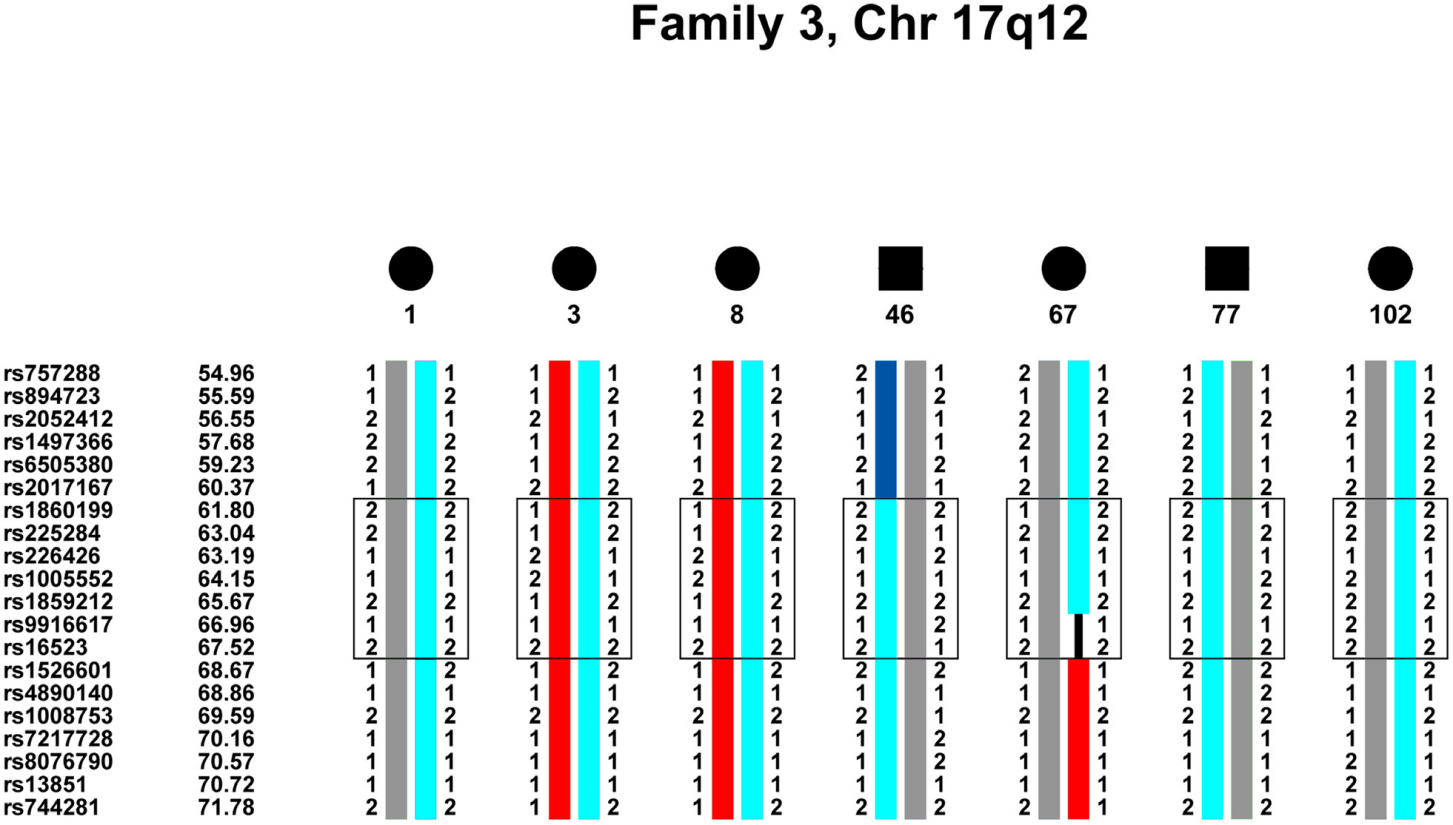
Haplotype of pedigree 3, chromosome 17q12. An approximate 6 cM region from rs1860199 (61.80 cM) to rs16523 (67.52) in chromosome 17q12 cosegregates IBD with all of the clinically and EEG affected members in the pedigree. The cosegregating haplotype is in light blue. The upper boundary is demarcated by a recombination in member 46 and the lower boundary by a recombination in member 67.

Haplotype analysis of the entire genome in pedigree 3 identified one definite cosegregating region, a 6 cM area in 17q12 bordered by rs1860199 and rs16523 (Fig. 5). However, we cannot rule out two other possible cosegregating regions in 1q32 and 13q14.2 because we do not have the haplotypes of “married ins” and parents of some epilepsy affected persons. This created regions of uninformative markers and an inability to determine the extent and borders of specific recombination sites in certain individuals. As such, we cannot rule out cosegregation with all clinical and EEG affected members in a 5 cM region in 1q32.1 from rs4351714 to rs14028 (Fig. S2), and a 0.5 cM region in 13q14.2 bordered by rs912278 and rs1427167 (Fig. S3). Of note, the cosegregating region on 13q14.2 overlaps the centromeric portion of the cosegregating 13q13.3–q31.2 region found in pedigree 2 and supports pedigree three's linkage region in 13q21.31 with *Z*_max_ of 2.04.

Performing haplotype analysis of the entire genome in pedigree 3, rather than just the areas suggested by linkage analysis, was integral in finding the one definite cosegregating region in 17q12. The cosegregating region in 17q12 and the possible region in 1q32.1 have no supporting linkage results from either individual or pooled pedigree analysis. This may appear confusing when results of haplotype analysis do not have supporting linkage results. Such occurs because the variance in LOD scores is not entirely based on the pattern of segregation in the family but also the informativeness of the markers in question (the number of heterozygous meioses). Since SNP markers were all biallelic, the number of allele combinations that can provide informative meiosis is reduced. However, SNPs do have the advantage that their density and distribution across the genome makes it much easier to impute an optimal haplotype due to the decreased likelihood of a double recombination occurring over a very small region. The segregating region in H3, for example, spanned approximately 55 cM, covered by 94 SNP markers. Of those 94 SNPs, using model #2, only 3 SNPs had LOD scores greater than 1.50 and 17 SNPs had LOD scores greater than 1.00 (Fig. S4). In the case of H5, all of the reported regions could only “potentially” cosegregate, due to the fact that only one “married‐in” was collected and haplotyped. This increased the space between informative markers where the parental haplotypes could not be definitively assigned.

Most importantly, whole‐genome haplotype analysis was essential in eliminating chromosome regions that had appeared significant for linkage and then failed to cosegregate in any of the pedigrees, such as 9q22.3 in pedigree 3, as well as 4q35.2 and 16p13.3 in the pooled linkage analysis of the three pedigrees. Linkage analysis combined with haplotype analysis did identify one locus in 2q21.2‐q31.1 for a CAE/JME gene and another locus in 13q13.3‐q31.2 for a JME/pA gene.

## Discussion

Juvenile myoclonic epilepsy remains an enigmatic epilepsy. Despite exhibiting varying seizure phenotypes within family members, differing frontocortical‐subcortical networks that underlie EEG polyspike waves, and changing prognoses for seizure remission, JME researchers continue to explain JME as a single disease (Koepp et al. 2014). In our present linkage analysis, supported by haplotypes that cosegregated with epilepsy affected members, we identified a locus in chromosome 2q21.2–2q31.1 that may contain a gene for CAE/JME in pedigree 1, and a locus in chromosome 13q13.3–13q31.2 that may contain a gene for JME/pA in pedigree 2. In pedigree 3, haplotypes identified a 6 cM region in 17q12, which may contain a gene for cJME. However, cosegregation of 13q14.2 and 1q32.1 cannot be ruled out in pedigree 3 because lack of haplotypes in “married ins” prevented definition of true borders of recombinations. These results, nonetheless, indicate there are three different JME‐causing genes in these three Honduras families, supporting the concept that there are several diseases underlying JME. JME is more correctly called juvenile myoclonic epilepsies.

Our results support the practice of analyzing JME pedigrees by proband subsyndrome rather than just JME alone. Our experience also confirms that large families are ideal for linkage analysis when phase known meioses across several generations can precisely determine haplotypes identical by descent, and lead to the identification of a Mendelian gene with major displacement.

Within the 2q21.2 to 2q31.1 candidate region for pedigree 1, various disease entities have been recognized, such as Dravet syndrome caused by de novo mutations in SCN1A (182389) in 2q24 (Krepischi et al. 2010), and a severe epilepsy resembling Dravet syndrome in 2q22.1 to 2q33.3 (Davidsson et al. 2008). Mutations in Zinc finger E‐box‐binding homeobox 2 in 2q22.3 and microdeletions in 2q21–q23 have been linked to epilepsy producing Mowat–Wilson syndrome (Garavelli et al. 2009). Deletions in 2q21.1 have also been linked to five families with attention‐deficit hyperactivity disorder with various developmental difficulties and epilepsy (Dharmadhikari et al. 2012).

The large cosegregating region from chromosome 13q13.3–q31.2, suggested by individual linkage and haplotyping results in pedigrees 2 and 3, as well as the pooled pedigree linkage results, contains several chromosome areas that have previously been implicated in various epilepsies. Idiopathic generalized epilepsies have previously been genetically linked to locus 13q31.3 (EPICURE Consortium et al. 2012), and IGEs along with the photoparoxysmal response have been implicated to be located in 13q13 and 13q31 (Tauer et al. 2005; Torniero et al. 2007; De Kovel et al. 2010a,b). A preferential predisposition for absence seizures has also been linked to 13q22–q31 (Hempelmann et al. 2006). Singular cases of epilepsy combined with other neurological disorders or mental retardation have been linked to 13q22 and to *SLC4A10*, or solute carrier family 4, sodium bicarbonate transporter, member 10 in 13q31 (Gurnett et al. 2008; Ribacoba et al. 2008). However, locus 13q14.2, a region possibly cosegregating with two of the Honduran pedigrees under investigation, has not been previously associated with epilepsy, but has been linked to visual migraine aura (Tikka‐Kleemola et al. 2010).

Pedigree 3 produced more varying results, with haplotypes definitely cosegregating in 17q12 and possibly cosegregating in 1q32.1 and 13q14.2. The 17q12 locus has been linked with generalized epilepsies and febrile seizures, as well as with migraines comorbid in Rolandic epilepsy (Sirén et al. 2010; Hardies et al. 2013; Addis et al. 2014). Locus 1q32.1 contains two genes previously implicated in epilepsy: *CAMSAP1L1* or calmodulin regulated spectrin‐associated protein family, member 2 found in a GWAS of Chinese epilepsy patients (Guo et al. 2012), and *SRGAP2* or SLIT‐ROBO Rho GTPase activating protein 2 in a single patient with early infantile epileptic encephalopathy and severe psychomotor disability (Saitsu et al. 2012).

There is a wide range of seizure phenotypes across JME families, and even within the same JME family. While our pedigrees were ascertained through a proband with a JME diagnosis, these probands exhibited three distinct subsyndromes of the disease based on the age at occurrence of each seizure phenotype and specific EEG pattern, the presence of myoclonias, generalized clonic‐tonic‐clonic or tonic‐clonic grand mal seizures and absence seizures, and the sequence of their appearance. The family members of these pedigrees exhibited a wide range of epilepsy phenotypes and did so with different patterns based on the proband's JME subsyndrome. Pedigree 1, whose proband had CAE/JME, had the highest number of family members with “absence epilepsy only”. There were no cases of classic JME without absence seizures in family members of pedigree 1 suggesting this family exhibits more of an absence syndrome. Pedigree 2, whose proband had JME/pA, had cases that were primarily “JME only” or “absence epilepsy only” in family members suggesting this family has a syndrome with mixtures of absence and JME. Pedigree 3, whose proband had cJME, had family members consisting mostly of JME cases and because none of the family members had “absence epilepsy only” this pedigree 3 is a true classic JME family. These observations coincide with previous reports that epilepsy diagnoses in family members vary by the proband's diagnosis, and that family member diagnoses are more likely to share epilepsy syndrome and seizure type with the proband (Winawer et al. 2003; Marini et al. 2004; Kinirons et al. 2008).

Because differences can be seen in the presentation and frequency of epilepsy phenotypes in family members of specific pedigrees based on the JME subsyndrome, it has been argued that the subsyndromes are actually different forms of epilepsy diseases that should not be analyzed together under the single diagnosis of JME. The three candidate chromosome loci, namely 2q21.2 to 2q31.1 for CAE/JME in pedigree 1, 13q13.3 to 13q31.2 for JME/pA in pedigree 2 and 17q12 for cJME in pedigree 3 support this concept.

We conducted linkage analysis using different diagnostic models to simulate varying levels of clinical heterogeneity within and among the pedigrees and assess the effects on the LOD score. Evidently, the highest LOD scores for each pedigree and the combination of pedigrees were produced under the most clinically heterogeneous diagnostic model #2, which included all forms of epilepsy plus the clinically asymptomatic members with EEG polyspike waves. Haplotype analysis of the entire genome for each pedigree was critical for determining which chromosome regions cosegregated and which regions coincided with the chromosome loci identified by linkage analysis. These results argue that clinical phenotypic heterogeneity within a pedigree will not inhibit analysis and can actually increase the LOD score. Despite having a proband diagnosis of JME, these results further support the notion that these pedigrees should not be combined, because we would have expected the cosegregating regions to overlap among the pedigrees and the combined LOD score to be closer to the higher ELOD score.

Despite our best efforts, our results are subject to limitations, which, in turn, advise caution in interpretations. First, is the unavailability of DNA due to deceased members and members unwilling to participate which is not uncommon in family‐based studies. We lacked DNA for deceased founders in all three pedigrees and for several “married‐in” individuals, most notably in pedigree 3. This prevented notations of phase known meioses, forcing imputations of haplotypes, and preventing definition of a single candidate region for pedigree 3. Second, is the caution in how our results should be interpreted in the overall context of the complex heredity of epilepsy. Linkage results of complex traits can be unreliable and inexact. Most analyses of the complex hereditary factors in epilepsy have often failed to disentangle heredity from environment. Usually a multifactorial or complex genetic model of polygenes acting on environment is inferred. Here, linkage and haplotype analysis in three large multigeneration kindreds with many matings and a large number of offspring with well defined epilepsy and EEG endophenotypes emphasize the role of locus heterogeneity and differing mutated genes in 13q13.3‐31.2 for JME with adolescent onset pyknoleptic absence, 2q21.2–q31.1 for CAE evolving to JME and 17q12 for classic JME. Definite understanding of the differing clinical epilepsy and EEG endophenotypes within these JME families will require identification of the many variants within the many specific epilepsy‐causing genes and their neurobiological disease mechanisms.

## Conflict of Interest

None of the authors has any conflicts of interests to disclose.

## Supporting information


**Figure S1.** Individual genome scans for all three pedigrees for each of the four diagnostic models.

**Figure S2.** Haplotype of pedigree 3, chromosome 1q32.1.

**Figure S3.** Haplotype of pedigree 3, chromosome 13q14.2.

**Figure S4.** 170 SNPs genotyped on chromosome 13 from Family 2.

**Table S1.** Simulated ELOD results for Honduran pedigrees.
