# Supplementary figures and images for: Chromosome loci vary by juvenile myoclonic epilepsy subsyndromes: linkage and haplotype analysis applied to epilepsy and EEG 3.5–6.0 Hz polyspike waves
Source: Mol Genet Genomic Med. 2016 Jan 23;4(2):197–210. doi: 10.1002/mgg3.195 (PMC4799870; doi:10.1002/mgg3.195)

Supplementary Figure S1. Results of genome scans of the 3 pedigrees for the 4 models.
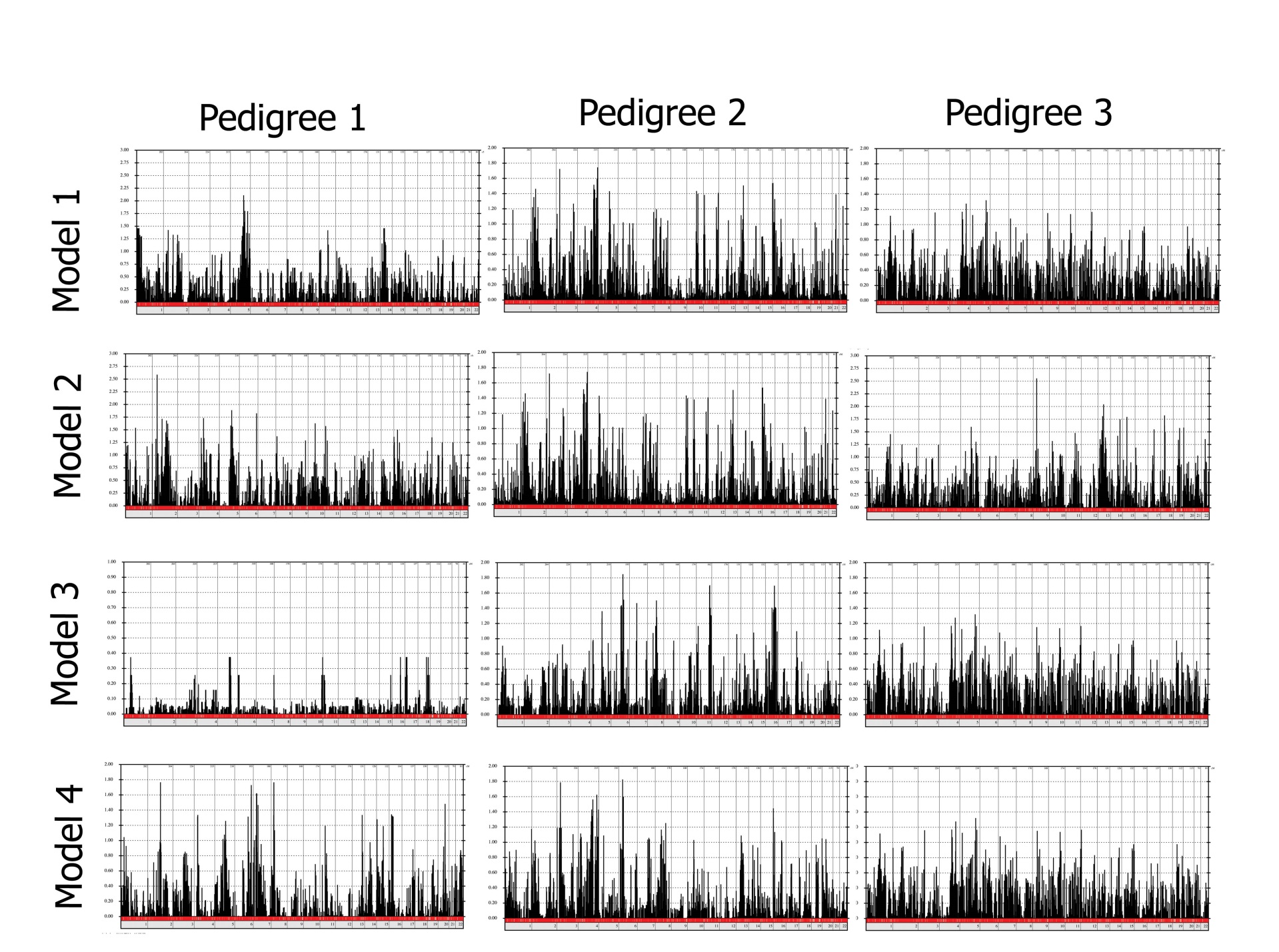

Supplement: Supplementary file 1 — Figure S1. Individual genome scans for all three pedigrees for each of the four diagnostic models. [file MGG3-4-197-s001.docx]

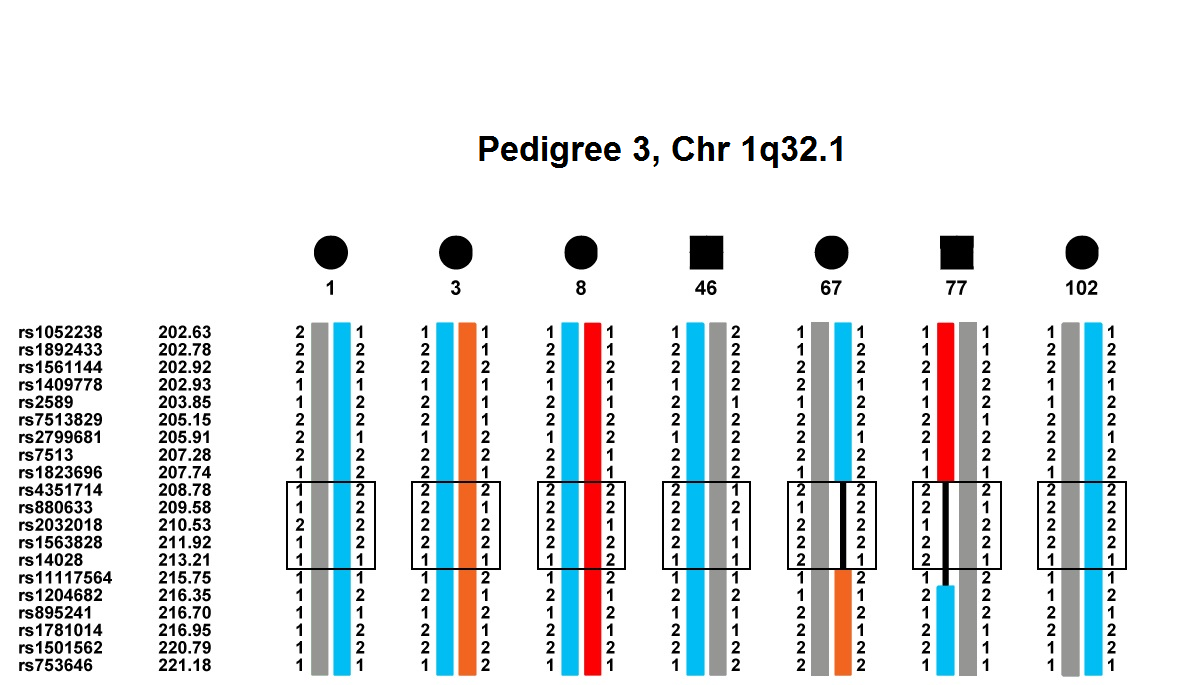

Supplement: Supplementary file 2 — Figure S2. Haplotype of pedigree 3, chromosome 1q32.1. [file MGG3-4-197-s002.tif]

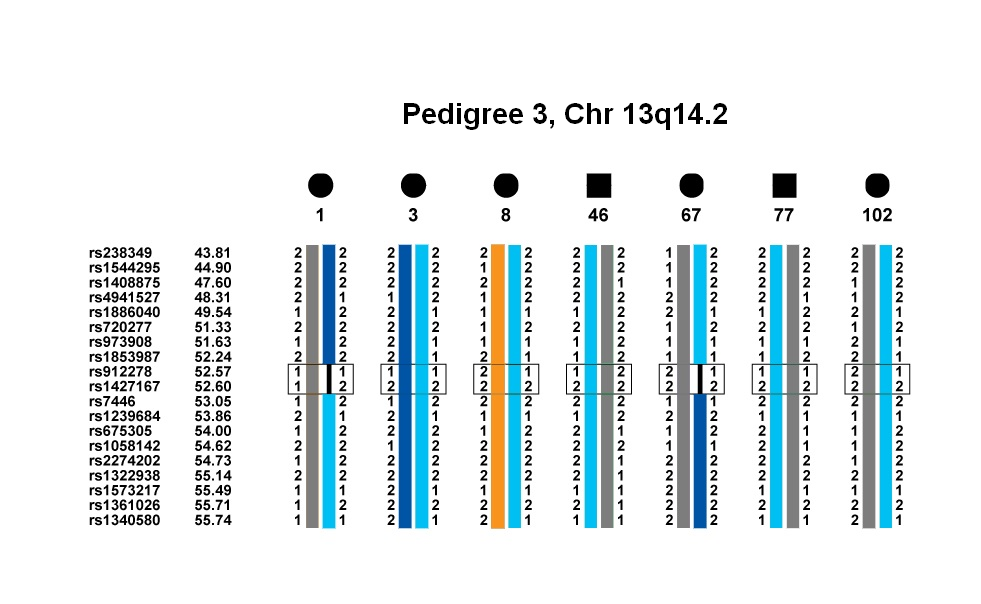

Supplement: Supplementary file 3 — Figure S3. Haplotype of pedigree 3, chromosome 13q14.2. [file MGG3-4-197-s003.tif]

**Supplementary Figure S4. SNP Marker Information and LOD-Score on Chr13 (Pedigree 2)**

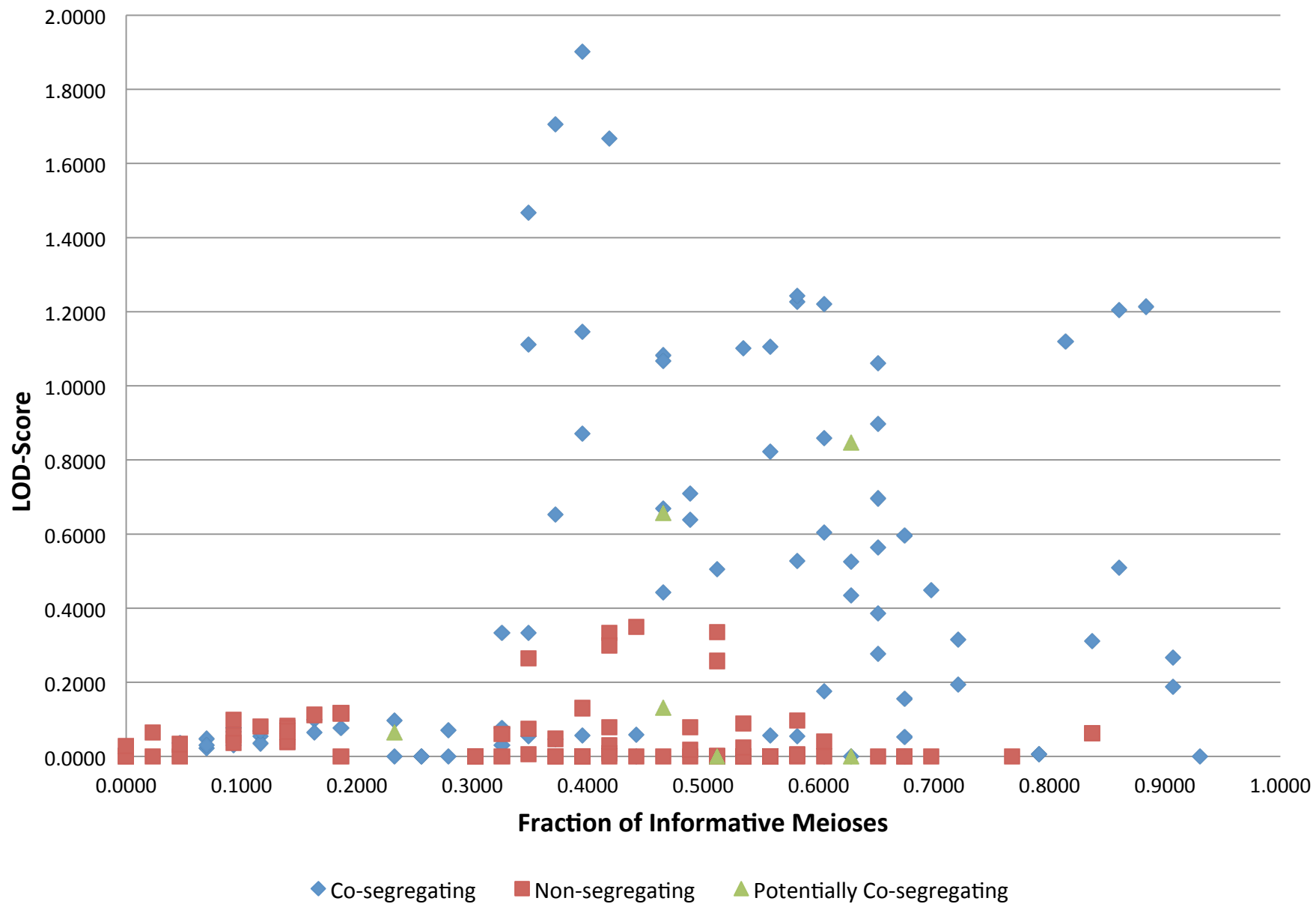

Supplement: Supplementary file 4 — Figure S4. 170 SNPs genotyped on chromosome 13 from Family 2. [file MGG3-4-197-s004.pdf]
