# Supplementary material for: Chromosome loci vary by juvenile myoclonic epilepsy subsyndromes: linkage and haplotype analysis applied to epilepsy and EEG 3.5–6.0 Hz polyspike waves
Source: Mol Genet Genomic Med. 2016 Jan 23;4(2):197–210. doi: 10.1002/mgg3.195 (PMC4799870; doi:10.1002/mgg3.195)
Supplement: Supplementary file 5 — Table S1. Simulated ELOD results for Honduran pedigrees. [file MGG3-4-197-s005.docx]

Supplement Table I. Simulated ELOD results for Honduran pedigrees.

|  | | Pedigree 1 | | | Pedigree 2 | | | Pedigree 3 | | | All Pedigrees | | |
| --- | --- | --- | --- | --- | --- | --- | --- | --- | --- | --- | --- | --- | --- |
|  |  | Avg ELOD | Min ELOD | Max ELOD | Avg ELOD | Min ELOD | Max ELOD | Avg ELOD | Min ELOD | Max ELOD | Avg ELOD | Min ELOD | Max ELOD |
| Diagnostic Model | #1. Clinically Affected | 0.422 | -1.776 | 2.734 | 0.925 | -2.584 | 3.852 | 0.615 | -1.657 | 2.813 | 1.909 | -2.092 | 6.756 |
|  | #2. Clinically Affected + EEG PSW | 0.902 | -1.715 | 3.628 | 1.073 | -2.526 | 4.170 | 0.606 | -1.767 | 3.000 | 2.625 | -1.818 | 8.069 |
|  | #3.  JME Affected | 0.003 | -0.118 | 0.375 | 0.625 | -2.356 | 3.087 | 0.329 | -1.614 | 1.675 | 1.002 | -2.213 | 4.632 |
|  | #4. JME Affected + EEG PSW | 0.306 | -1.049 | 3.067 | 0.861 | -2.607 | 3.994 | 0.342 | -1.801 | 1.794 | 1.451 | -1.725 | 5.749 |

Estimated LOD (ELOD) scores were simulated for each diagnostic model under each pedigree and under the combination of pedigrees. Family members that were considered affected under each model are as follows: (model #1) all members affected with clinically symptomatic epilepsy, (model #2) all members affected with clinically symptomatic epilepsy and members with the EEG polyspike wave (PSW) trait, (model #3) all members affected with JME, and (model #4) all members affected with JME and members with the EEG PSW trait. ELOD scores were simulated using FastSLink v2.51 in the EasyLinkage Plus v5.08 package using an autosomal dominant model with 70% penetrance, disease allele frequency of 0.01, penetrance of the homozygote non-disease genotype of 0.001, and 1000 replications.
