# Supplementary material for: Chromosome loci vary by juvenile myoclonic epilepsy subsyndromes: linkage and haplotype analysis applied to epilepsy and EEG 3.5–6.0 Hz polyspike waves
Source: Mol Genet Genomic Med. 2016 Jan 23;4(2):197–210. doi: 10.1002/mgg3.195 (PMC4799870; doi:10.1002/mgg3.195)
Supplement: Supplementary file 6 [file MGG3-4-197-s006.docx]

**SUPPLEMENT FIGURE LEGENDS**

**Supplement Figure 1:** Individual genome scans for all three pedigrees for each of the four diagnostic models.  Two-point Lod scores are depicted on the 'Y' axis and Chromosome is on the 'X' axis.

**Supplement Figure 2. Haplotype of pedigree 3, chromosome 1q32.1.** An approximate 6 cM region from rs1823696 (207.74 cM) to rs14028 (213.21) in chromosome 1q32.1 potentially co-segregates IBD with all of the clinically and EEG affected members in the pedigree. The uncertainty of IBD co-segregation derives from a small region of overlap when taking into account the uninformative markers that determine the recombinations in 77 and 67 (the upper and lower boundaries, respectively). The potentially co-segregating haplotype is in light blue.

**Supplement Figure 3. Haplotype of pedigree 3, chromosome 13q14.2.** An approximate 0.5 cM region from rs912278 (52.57 cM) to rs1427167 (52.60) in chromosome 13q14.2 potentially co-segregates IBD with all of the clinically and EEG affected members in the pedigree. The uncertainty of IBD co-segregation derives from the uninformative markers that cannot determine the exact position of recombinations in members 1 and 67 (the upper and lower boundaries respectively) and, therefore, they potentially overlap. The potentially co-segregating haplotype is in light blue. This same region is demarcated within the co-segregating region of pedigree 2 in Figure 4.

**Supplement Figure 4:** 170 SNPs genotyped on chromosome 13 from Family 2.  A scatter plot of the fraction of informative meioses (heterozygous parental genotype) and Linkage LOD-Score.  Red squares are SNPs that were determined not to co-segregate among all of the affected members of the family by haplotyping analysis.  Blue diamonds are SNPs in the approximately 44 cM that co-segregates with all of the Affected members of the family.  Green triangles are the SNPs in the flanking regions where co-segregation cannot be definitively determined.
